# circRNA circ_0055724 Inhibits Trophoblastic Cell Line HTR-8/SVneo's Invasive and Migratory Abilities via the miR-136/N-Cadherin Axis

**DOI:** 10.1155/2022/9390731

**Published:** 2022-06-22

**Authors:** Xiaohong Xu, Hong Teng

**Affiliations:** Department of Obstetrics and Gynecology, The Second Hospital of Jilin University, No. 218 Ziqiang Street, Changchun, 130000 Jilin, China

## Abstract

Preeclampsia (PE) is one of the major causes of morbidity and mortality in pregnancy. According to recent research, circular RNAs (circRNA) may act as sponges for microRNAs (miRNAs) and modulate gene expression. Low expression of hsa_circ_0055724 (circ_0055724) in PE tissues was recently reported in literatures. However, its mechanism and function have not been reported. Therefore, we were committed to investigating the role and mechanism of circ_0055724 in PE. Our study first verified the low expression of circ_0055724 in PE tissues. Overexpression or knockdown of circ_0055724 enhances/weakens the trophoblast cell survival, migration, and invasion. Furthermore, CircInteractome predicted the binding sites of circ_0055724 and miR-136, while Starbase predicted miR-136 targeted N-cadherin. Luciferase reporter gene assay confirmed that circ_0055724 directly interacts with miR-136 and miR-136 directly interacts with N-cadherin. More results indicated that high expression of miR-136 and low expression of N-cadherin appeared in PE. Increased expression of circ_0055724 resulted in decreased miR-136 but increased N-cadherin expression. Hence, circ_0055724 and N-cadherin were positively correlated, while circ_0055724 and miR-136 had a negative correlation. In terms of mechanism, circ_0055724 may induce the expression of N-cadherin and regulate the proliferation, migration, and invasion of trophoblast cells through decreasing miR-136, which can be a promising biomarker for early diagnosis and prognosis of patients with PE.

## 1. Introduction

PE is one of the most feared complications of pregnancy, which is characterized by a new onset of hypertension with evidence of maternal organ or uteroplacental dysfunction or proteinuria after 20 weeks of gestation [1]. These disorders remain one of the leading causes of maternal and perinatal morbidity and mortality [2]. A recent meta-analysis of 291, 247 adolescents worldwide since 1969 has shown that preeclampsia/eclampsia had an overall prevalence rate of 6.7% [3]. PE is also associated with adverse fetal effects, including limitation of fetal growth, preterm delivery, placental abruption, foetal stress, and intrauterine death [1, 4, 5].

Etiology of PE remains incompletely understood although rigorous efforts and a variety of mechanisms have been made to contribute to investigating the pathogenesis of this complication. Various studies have suggested that both maternal and placental-derived factors might involve. Maternally driven PE might result from preexisting maternal disorders that predispose the mother to cardiac complications including hypertension [6]. These disorders may include hypertension, renal disease, overweight, and diabetes that might not be evident before pregnancy. In contrast, placental-derived PE might result from reduced placental perfusion caused by shallow trophoblast invasion and unconverted narrow spiral arteries. During the early stage of pregnancy, interstitial trophoblasts anchor the embryo to the placenta, while endovascular extravillous cytotrophoblast (eEVT) migrate to the spiral arteries of the placenta and invade/replace the endothelial cells lining these vessels [7]. EVI also takes part in the loosening of maternal spiral arteries by the degradation of the muscular coat. This eventually results in the formation of low-resistance high-capacitance vessels that regulate the maternal blood flow to the placenta (conversion) [8]. Defects in remodelling of spiral arteries due to insufficient trophoblast migration and invasion cause high-resistance low-capacitance vessels. The resulting hypoxia might then translate into endothelial injury, eventually leading to maternal hypertension and proteinuria [8, 9].

Not all human genome sequences encode proteins, and noncoding RNA could account for 95% of the total RNA transcribed from the eukaryotic genome [10]. These noncoding RNAs (ncRNAs) are classified as PIWI-interacting RNA, small nucleolar RNA, long noncoding RNA (lncRNA), miRNA, and circRNA [11]. With the advances in cell biology and bioinformatics, the role of these ncRNAs is increasingly being recognized in the genetic regulation and development of human disease. circRNAs are molecules with a closed-loop structure, unlike linear RNA, and make up a significant proportion of the ncRNA family [12]. circRNAs, resistant to RNases, have broad presence and expression, and their expression appears to be conserved across mammals [11].

Long after their discovery in 1976, circRNAs have attracted intense interest during the past few years [13]. Among various other roles, circRNA might act as miRNA sponges and thereby regulate gene transcription. The regulatory role of circRNAs in different disorders by acting as miRNA sponge has been reported in various studies [14–17]. Especially, circRNA can be used as a new biomarker to diagnose and treat preeclampsia early, such as circRNA-0004904, circRNA-0001855, and circPAPP-A [18]. Moreover, Hu et al. revealed that circ_0055724 can act as ceRNA expression profiling to identify in PE as a potential novel blood biomarker for early PE [19], but this study did not study the intrinsic molecular regulation mechanism of circ_0055724 in PE. However, to our knowledge, no study is yet available that describes the precise role and the mechanism of function of circ_0055724 in PE.

Hence, our study was aimed at examining the function and elucidating the underlying mechanisms of circ_0055724 in PE. Our results validated that circ_0055724 is specifically expressed in preeclampsia, and increased expression of circ_0055724 can promote the proliferation, migration, and invasion of trophoblast cells HTR-8/SVneo. In terms of mechanism, circ_0055724 may affect the expression of N-cadherin and regulate the proliferation, migration, and invasion of trophoblast cells through sponge miR-136, indicating that this pathway is a novel target for developing therapeutic strategies to treat PE.

## 2. Materials and Methods

### 2.1. Clinical Samples

The placenta tissues were obtained from patients with severe PE (*n* = 40) between 28 and 36 years age at 28 to 36 weeks of pregnancy and normal pregnant women at 30 to 38 weeks of pregnancy whose maternal age at delivery is 28 to 35 (*n* = 40) at the Second Hospital of Jilin University from April 2015 to August 2020. The time between placenta birth and sample collection is about 2 months, and the diagnosis criteria for severe PE were as follows: systolic pressure ≥ 160 mmHg and/or diastolic pressure ≥ 110 mmHg on at least two occasions with 6 h apart, accompanied by severe proteinuria (>5 g/24 h urine specimen or 3+ on ≥2 random samples collected 4 h apart). Normotensive pregnancy was characterized by not having PE or other complications, including premature rupture of membranes, fetal anomalies, cardiac disease, and maternal history of hypertension as well as smoking. All the participants were informed about the study, and their written informed consent was obtained. The samples were maintained at the Second Hospital of Jilin University. Immediately after collection, the samples were frozen and stored at -80°C for further use. All the protocols of the study were reviewed and approved by the ethical committee of the Second Hospital of Jilin University and were in accordance with the international standards for human experimentation.

### 2.2. Cell Culture

Previously reported procedures for the culture of HTR-8/SVneo cells after three passages were used [20]. Cells were cultured in Roswell Park Memorial Institute (RPMI-1640) medium (Thermo Fisher Scientific, USA) supplemented with 10% fetal bovine serum (FBS) and 1% penicillin/streptomycin (Thermo Fisher Scientific, USA). The cells were maintained under standard laboratory conditions (5% CO_2_, at 37°C). Lipofectamine 2000 (Invitrogen, USA) was used for the transfection of the cells with different reagents according to the manufacturer's protocols.

### 2.3. Cell Transfection

The design and synthesis of siRNA targeted to circ_0055724, inh-miR-136, and mimics-miR-136, as well as their NC negative controls (mimics-NC, inh-NC), were separately designed by Genechem (Shanghai, China). The open reading frame of circ_0055724 was inserted into the expression vector pcDNA 3.1(+) (Sigma, USA) to overexpress circ_0055724, which was also conducted by Genechem (Shanghai, China). An empty vector (pcDNA3.1(+)) was used as a negative control for circ_0055724 overexpression. The HTR-8/SVneo cells were transfected with indicated constructs using the Lipofectamine 2000 (Invitrogen, USA) reagent at 37°C and collected for 72 h after transfection for the subsequent experimentation. Following transfection, qRT-PCR was performed to investigate the transfection efficiency. The sequences of si-NC, si-circ_0055724, mimics-NC, inh-NC, inh-miR-136, and mimics-miR-136 were as follows:

- si-circ_0055724#1: 5′-GCCTTGACGCATTTTTGATCT-3′
- si-circ_0055724#2: 5′-ACCACCAGCCTTGACGCATTT-3′
- si-NC: 5′-CAACAAGATGAAGAGCACCAA-3′
- miR-136 mimics: 5′-ACUCCAUUUGUUUUGAUGAUGG-3′
- mimics-NC: 5′-TTCTCCGAACGTGTCACGT-3′
- miR-136 inhibitors: 5′-CCAUCAUCAAAACAAAUGGAGU-3′
- Inhibitor-NC: 5′-UUCUCCGAACGUGUCACGUTT-3′
- si-N-Cadherin: 5′-UGAAGAUACACACAUAACGCC-3′
- si-NC: 5′-CAACAAGATGAAGAGCACCAA-3′

### 2.4. CircInteractome

As a web tool, CircInteractome is often used to explore different circRNAs and their target miRNAs or proteins based on the Targetscan algorithm [21]. Here, this tool was used for the prediction of the binding site of circ_0055724 based on bioinformatics analysis using the CircInteractome database (https://circinteractome.nia.nih.gov/).

### 2.5. Transwell Assay

This assay was used to detect the number of invasive and migrating cells following different interventions [22]. Briefly, for invasion assay, 1 × 10^5^ cells were suspended in 200 *μ*L of medium without serum in the upper chamber (8 *μ*m pore size) with a porous membrane that contained Matrigel solution (50 mg/L, dilution ratio is 1 : 8, BD, USA). The lower chamber was immersed into a well that contained 600 *μ*L of cell culture medium with 10% serum. The cells were treated with siRNA for 24 h at 37°C. Cotton swabs were used to remove the noninvasive cells from the upper membrane surface. The invasive cells that were present at the surface of the lower membrane were fixed with 4% formaldehyde followed by staining with 0.1% solution of crystal violet. The cells were observed under an Inverted Nikon Eclipse Ti microscope. A similar procedure was used for the cell migration assay except that the chambers did not contain Matrigel.

### 2.6. Cell Counting Kit-8 (CCK-8) Assay

Previously reported procedures were used with modification [20]. For this assay, 2 × 10^3^ cells (per well) were seeded onto 96-well plates (100 *μ*L). The incubation was done for different time intervals 0 h, 24 h, 48 h, and 72 h. Following the incubation, 10 *μ*L CCK-8 solution (Dojindo, Japan) was added followed by incubation at 37°C in dark for a period of 1 h. The absorbance was detected at a wavelength of 450 nm using a microplate reader (Synergy H4 Hybrid Reader, BioTek, Winooski, USA).

### 2.7. Quantitative Reverse Transcription Polymerase Chain Reaction (qRT-PCR)

Total RNA was extracted from the preeclampsia and normal placental tissue samples using TRIzol (Invitrogen, USA). The manufacturer's protocol was used to reverse transcribe the isolated RNA to cDNA using PrimeScrip™ RT Master Mix (Thermo Fisher Scientific, USA) for 15 min at 37°C. SYBR Green PCR Master Mix (Thermo Fisher Scientific, USA) was used to analyse the gene expression using the 2^-*ΔΔ*Ct^ method. NanoDrop-2000 spectrophotometers (Thermo Fisher Scientific, USA) were used to check the concentration and purity of RNA (A260/A280 ratio of all samples was between 1.9 and 2.1). The primer sequence used was as follows:

- miR-136: F: 5′-ACACTCCAGCTGGGACTCCATTTGTTTTG-3′, R: 5′-CTCAACTGGTGTCGTGGAGTCGGCAATTCAGTTGAGTCCATCAT-3′
- circ_0055724: F: 5′-AGCCACAGAAATAAAGGATGGAGA-3′, R: 5′-TCTGAGAAGACACTGGATTGCTA-3′
- N-cadherin: F: 5′-GGTGGAGGAGAAGAAGACCAG-3′, R: 5′-GGCATCAGGCTCCACAGT-3′
- GAPDH: F: 5′-ACGGGAAGCTCACTGGCATGG-3′, R: 5′-GGTCCACCACCCTGTTGCTGTA-3′
- U6: F: 5′-CTCGCTTCGGCAGCACA-3′, R: 5′-AACGCTTCACGAATTTGCGT-3′

### 2.8. Colony Formation Assay

This assay was conducted to access the colony forming ability of the cells. Hence, 500 cells per well were seeded onto 6-well plates and grown for 14 days. Then, the cells were fixed with paraformaldehyde (4%) flowed by 30 min and stained with crystal violet (Beyotime, China) solution (0.4%). The colonies were counted under an inverted microscope.

### 2.9. Luciferase Reporter Assay

Through CircInteractome, we found the miR-136 binding site in circ_0055724. Therefore, luciferase reporter assay was used for confirming the interaction. For this assay, the wild type of circ_0055724 (circ_0055724 WT) and CDH2 (CDH2 WT-1, WT-2) and mutant type (circ_0055724 Mut, CDH2 Mut-1, Mut-2) reporter vectors were established by Beijing TransGen Biotech Co. (Beijing, China). 5 × 10^4^ cells were seeded for 24 h in 24-well plates. Lipofectamine 2000 (Invitrogen, USA) was used for the transfection/cotransfection of reporter plasmids with either miR-136 mimics or negative control of cells for 48 h at 37°C by following the manufacturer's guidelines. Subsequently, the luciferase activity was performed by using the luciferase assay kit (Promega, USA) according to the manufacturer's guidelines. Dual luciferase activities were measured by a microplate reader (Synergy H4 Hybrid Reader, BioTek, Winooski, USA) [23].

### 2.10. Pull-Down Assay

Previously reported procedures were used to biotinylate circ_0055724 and negative control (NC) to bio-circ_0055724 and bio-NC according to its instruction and transfected into l × 106 cells at a final concentration of 50 nM for 48 h before harvest [24]. Next, 0.7 mL lysis buffer (5 mM MgClz, 100 mM KCl, 20 mM Tris (pH 7.5), and 0.3% NP-40) and a complete protease inhibitor cocktail (Roche Applied Science, IN) were added into the cell pellets. Subsequently, the cells were washed with PBS and qRT-PCR was performed for the quantification of bound RNA.

### 2.11. Western Blot Analysis

Radio Immunoprecipitation Assay (RIPA) buffer with added protease inhibitors (Sigma, USA) was used to cause cell lysis, and the total protein concentration was determined with BCA Protein Assay Kit by standard curve method (Beyotime, China). This was followed by protein separation by SDS-PAGE, and the separated proteins were transferred to the PVDF membrane (Millipore, USA). After 1 h of blocking with 5% nonfat milk, the membranes were incubated with primary antibodies N-cadherin (Cell Signaling Technology, Danvers, MA), diluted 1 : 1000, and GAPDH (Abcam, Cambridge, UK), diluted 1 : 1,000 at 4°C overnight. At the end of incubation, the membranes were washed followed by the addition of secondary antibodies according to the host of each primary antibody. Blots were visualized by Immobilon Western Chemiluminescent HRP Substrate Kit (Millipore), and the protein bands were quantified by using ImageJ software (USA).

### 2.12. Statistical Analysis

GraphPad prism software version 6 was used to perform the statistical analyses. Student's *t* test was used for two groups while the statistical analysis of more than two groups was done by using one-way ANOVA with Tukey's post hoc test. *p* values less than 0.05 were considered a statistically significant difference. The assays were run in triplicate, and the data were presented as mean± SD.

## 3. Results

### 3.1. circ_0055724 Is Downregulated in Placental Tissues from PE Women

**Figure 1:**
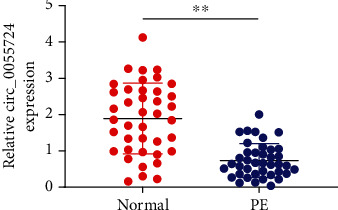
circ_0055724 was downregulated in placental tissues from pregnant women. The expression level of circ_0055724 in 40 normal tissues and 40 PE tissues was detected by qRT-PCR. The difference was statistically significant (*p* < 0.001).

qRT-PCR analysis indicated that placental tissue from PE women had a significantly low expression level of circ_0055724 than that from normal women (*p* < 0.001). In other words, circ_0055724 was downregulated in the placental tissue during preeclampsia (Figure 1).

### 3.2. circ_0055724 Increases Trophoblast Cell Viability, Invasion, and Migration

**Figure 2:**
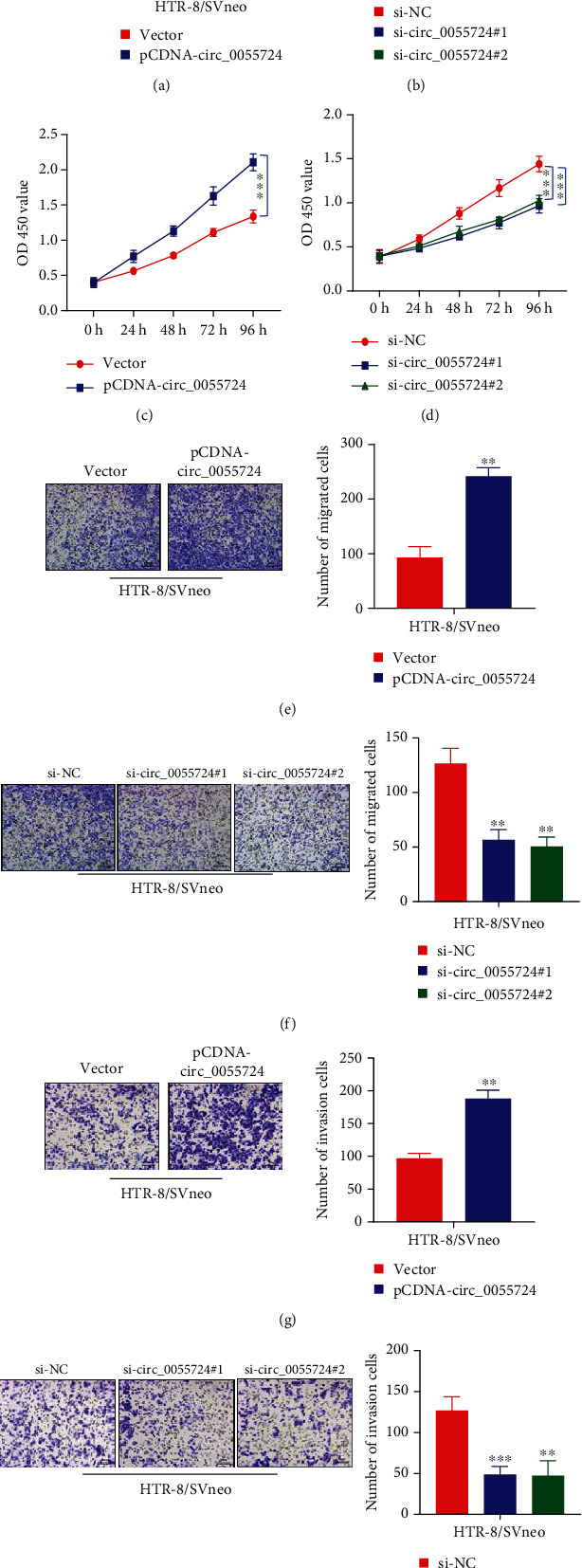
Effects of circ_0055724 on trophoblast proliferation, invasion, and migration. (a) Circ_0055724 was overexpressed in HTR-8/SVneo cells, and the overexpression efficiency was detected by qRT-PCR. (b) Knockdown of circ_0055724 in the HTR-8/SVneo cells and the qRT-PCR method was used to detect knockdown efficiency. (c, d) Represent the results of the CCK-8 method that was used to detect the light absorption values of HTR-8/SVneo cells at 450 nm wavelength at 0 h, 24 h, 48 h, 72 h, and 96 h in different groups. (e, f) Represent the number of migrating HTR-8/SVneo cells detected by Transwell assay after overexpression or silencing of circ_0055724, respectively. (g, h) Represent the number of invasive cells after overexpression or silencing of circ_0055724, respectively. ^∗∗^*p* < 0.001.

To understand the role of circ_0055724 in trophoblast cell proliferation, migration, and invasion, circ_0055724 was either overexpressed in HTR-8/SVneo cells through transfection or downregulated by si-circ_0055724#1 and si-circ_0055724#2. The overexpression or si-knockdown of circ_0055724 in HTR-8/SVneo cells was confirmed by qRT-PCR (Figures 2(a) and 2(b)). It was demonstrated that HTR-8/SVneo cells that overexpressed the circ_0055724 had significantly greater (*p* < 0.001) cell viability than that in the controls (Figure 2(c)). In contrast, si-knockdown of circ_0055724 resulted in significantly decreased (*p* < 0.001) viability of these cells (Figure 2(d)). Along the same line, overexpression of circ_0055724 was associated with remarkably increased (*p* < 0.001) ability of HTR-8/SVneo cells to migrate (Figure 2(e)) and invade (Figure 2(g)). Contrary to this, the knockdown of circ_0055724 significantly reduced (*p* < 0.001) the number of migrating (Figure 2(f)) and invading HTR-8/SVneo cells (Figure 2(h)).

### 3.3. miR-136 Is a Target of circ_0055724 in Trophoblast Cells

**Figure 3:**
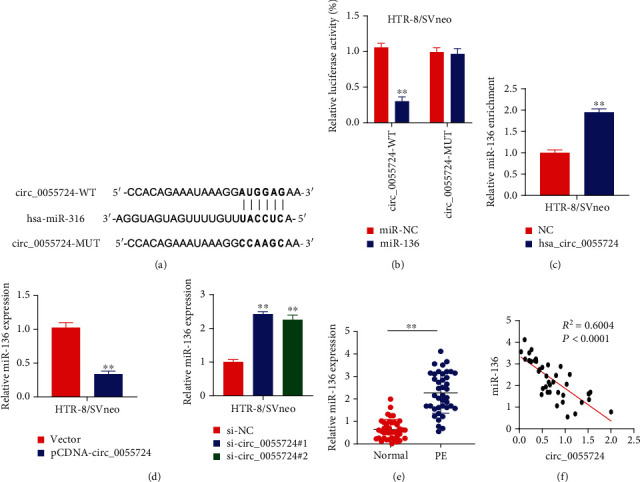
miR-136 was a target of circ_0055724 in trophoblasts. (a) Binding site of hsa_circ_0055724 on miR-136 was predicted by CircInteractome, an online bioinformatics tool. (b) Luciferase reporter gene assay revealed the inhibitory ability. (c) Interaction between biotinylated hSA_circ_0055724 and miR-136 was shown by RNA pull-down assay. (d) Represents the expression level of miR-136 in hsa_circ_0055724 cells detected by qRT-PCR after overexpression or knockdown of hsa_circ_0055724, respectively. (e) Represents the expression level of miR-136 in 40 normal tissues and 40 PE placental tissues detected by qRT-PCR. (f) Correlation analysis of hsa_circ_0055724 and miR-136 in PE placental tissue showed negative correlation. ^∗∗^*p* < 0.001.

The bioinformatics tool CircInteractome predicted that miR-136 shared the binding site of circ_0055724 (Figure 3(a)). Luciferase reporter gene assay was performed between circ_0055724 WT and circ_0055724 MUT groups. It was indicated that the overexpression of miR-136 was associated with significantly lower luciferase activity than controls in WT HTR-8/SVneo cells but not in the circ_0055724 MUT group (Figure 3(b)). In addition, it was clarified during the RNA pull-down assay that bio-circ_0055724 significantly enriched miR-136 than bio-NC in HTR-8/SVneo cells (Figure 3(c)). Quite interestingly, overexpression of circ_0055724 in HTR-8/SVneo cells was associated with significantly decreased miR-136 expression while circ_0055724 knockdown by si-circ_0055724#1 and si-circ_0055724#2 significantly increased miR-136 expression than the respective controls (Figure 3(d)). To confirm the physiological relevance of our findings, we conducted qRT-PCR on tissue samples from PE and normal women. It was corroborated that the placental tissue samples from PE women had significantly increased miR-136 expression than those from the normal controls (Figure 3(e)). In accordance, a negative correlation was observed between the expression level of miR-136 and circ_0055724 (*p* < 0.0001, *R*^2^ = 0.6004).

### 3.4. N-Cadherin Is a Direct Target miR-136

**Figure 4:**
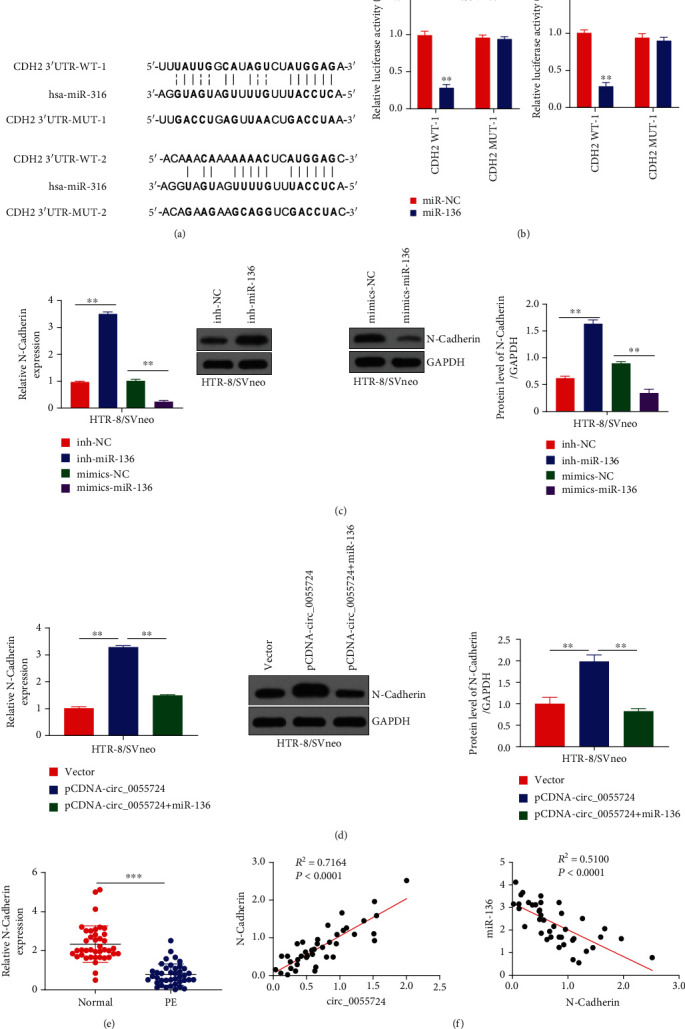
N-cadherin is a direct target of miR-136. (a) The binding sites of N-cadherin (gene CDH2) and miR-136 was predicted by Starbase, an online bioinformatics tool. (b) Represents the results of luciferase reporter gene experiment carried out in HTR-8/SVneo cells. (c) Compared with miR-NC, overexpression of miR-136 could inhibit luciferase activity in cells, and the inhibition effect disappeared after mutation of the predicted N-cadherin binding site. mRNA and protein expression levels of N-cadherin in miR-136 knockdown or overexpressed HTR-8/SVneo cells were detected by qRT-PCR and WB methods. mRNA and protein expression levels of N-cadherin were increased or decreased by miR-136 knockdown or overexpression, respectively. qRT-PCR and WB methods were used to detect the expression levels of N-cadherin mRNA and protein in HTR-8/SVneo cells in different groups (vector, circ_0055724, circ_0055724+ miR-136). (d) Overexpression of hsa_circ_0055724 increased the expression levels of N-cadherin mRNA and protein, and the expression levels of N-cadherin partially decreased after cotransfection with miR-136. (e) Represents the mRNA expression of N-cadherin in 40 normal tissues and 40 PE tissues detected by qRT-PCR. (f) Correlation analysis of hsa_circ_0055724, miR-136, and N-cadherin expression in PE. ^∗∗^*p* < 0.001.

Starbase analysis predicted that miR-136 had two binding sites on N-cadherin (expressed by the CDH2 gene).  Figure 4(a) indicates the sequences of the two binding sites (CDH2 3′UTR-WT-1, CDH2 3′UTR-WT-2) that act as the target of miR-136, along with the mutant gene sequence (CDH2 3′UTR-Mut-1, CDH2 3′UTR-Mut-2), respectively. In addition, the HTR-8/SVneo cells that overexpressed miR-136 had significantly reduced activity during the luciferase assay than NC-miR-136 and the inhibition effect disappeared after the mutation of the predicted binding sites (Figure 4(b)). First of all, qRT-PCR was performed to detect the expression of miR-136 in HTR-8/SVneo cells transfected with mimics-miR-136 and inh-miR-136 (Figures S1A, S1B). The results have shown that miR-136 exhibited a significantly increased expression in cells transfected with mimics-miR-136 but represented a decreased expression in cells transfected with inh-miR-136. It indicated that the transfection was a success. After that, the knockdown of miR-136 was associated with increased expression of N-cadherin while overexpression resulted in the decreased N-cadherin expression than the respective controls, as indicated by qRT-PCR and western blot analysis in HTR-8/SVneo cells (Figure 4(c)). Overexpression of circ_0055724 increased the expression levels of N-cadherin mRNA and protein, and the expression levels of N-cadherin partially decreased after cotransfection with miR-136, with a statistically significant difference (*p* < 0.01) (Figure 4(d)). To know the physiological significance of these findings, we conducted qRT-PCR analysis in PE and control tissue samples. It was found that N-cadherin expression was significantly reduced (*p* < 0.001) in PE placental tissue samples as compared to that in the normal control (Figure 4(e)). Hence, a positive correlation was detected between circ_0055724 (*R*^2^ = 0.7164, *p* < 0.0001) and N-cadherin while miR-136 and N-cadherin were negatively correlated with each other (*R*^2^ = 0.5100, *p* < 0.0001) (Figure 4(f)).

### 3.5. circ_0055724 Mediates Trophoblast Invasion and Migration via the miR-136/N-Cadherin Axis

**Figure 5:**
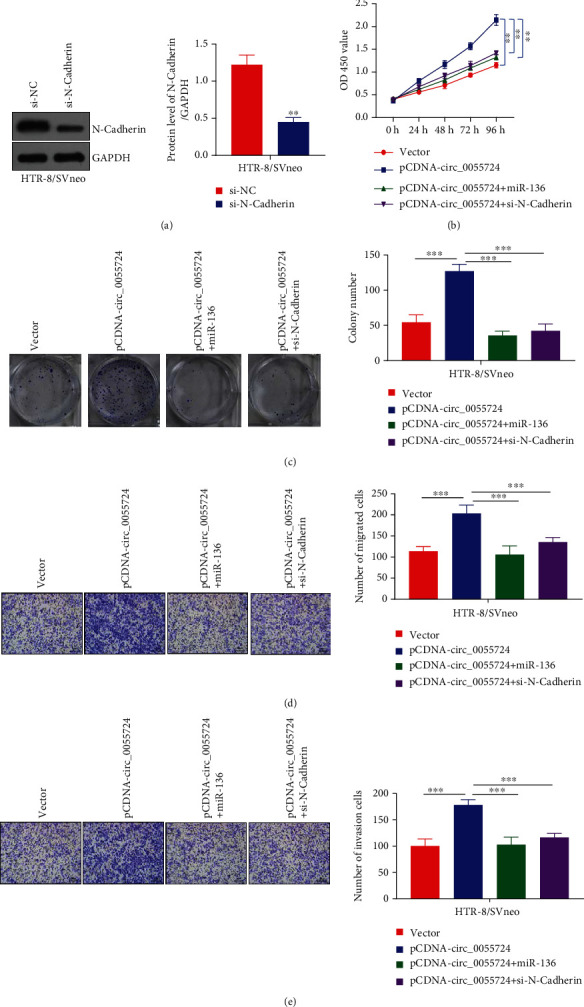
circ_0055724 mediated trophoblast invasion and migration via the miR-136/N-cadherin axis. (a) The WB method was used to detect the low level of N-cadherin in HTR-8/SVneo cells. (b) The CCK-8 method was used to detect the light absorption values of HTR-8/SVneo cells at 450 nm at 0 h, 24 h, 48 h, 72 h, and 96 h in different groups. (c) The clonal formation experiment was conducted to count the number of colonies and thereby estimate the colony forming capacity of the HTR-8/SVneo cells. (d) Cell migration ability of HTR-8/SVneo cells in different groups was detected by Transwell assay. (e) Invasion ability of HTR-8/SVneo cells in different groups is represented and determined by Transwell assay. ^∗∗^*p* < 0.001; ^∗∗∗^*p* < 0.0001.

si-N-cadherin was used to reduce the protein expression of N-cadherin in HTR-8/SVneo cells, and this low level of N-cadherin was confirmed through qRT-PCR western blot experiments (Figure 5(a)). It was demonstrated that the overexpression of circ_0055724 was associated with significantly higher cell viability than either of vector control, circ_0055724+miR-136 coexpression, or circ_0055724 overexpression but with N-cadherin silencing (Figure 5(b)). The overexpression of circ_0055724 also enhanced the clonal formation capacity, cell migration, and invasion ability of HTR-8/SVneo of cells (Figures 5(c)–5(e)) than the vector control. In contrast, circ_0055724+miR-136 coexpression or circ_0055724 overexpression but with N-cadherin silencing resulted in a nonsignificant difference in colony formation capacity, migration, and invasion ability of HTR-8/SVneo of cells than vector control (Figure 5(c)–5(e)).

## 4. Discussion

In the present study, placental tissue samples from 40 normal and 40 PE placentas were used and it was observed that the PE placental tissue samples had significantly low expression of circ_0055724, which has been previously corroborated by Hu et al. [19]. We used CircInteractome, an online computational tool, to predict the binding sites of circRNAs, on miRNAs using the Targetscan prediction tool [21]. In recent years, it has been reported that circRNAs functioned as miRNA “sponges” that can competitively inhibit miRNA activity [25]. miRNAs are small nucleotide molecules, with an average length of 21-23 nucleotides, which play an important role in the regulation of gene expression by guiding Argonaute proteins to specific mRNA [26]. In most cases, it involves the targeting of the 3′ untranslated region of the mRNA and results in the mRNA repression and degradation. Hence, miRNAs may play their role in gene silencing by inhibiting/degrading mRNA [27]. The role of miRNAs in the human pathologies is increasingly being recognized, particularly in cancers [28, 29] and some miRNAs have even been identified as biomarkers of certain diseases [30–33]. In a recent report, Motawi et al. have shown that miR-136 was increased in preeclampsia and could serve as an early circulating biomarker in the circulation [21]. Similar findings were also previously reported by Ji et al. [34]. CircInteractome predicted that circ_0055724 expressed the binding site for miR-136 and many other target genes, such as miR-1238, miR-1243, miR-1253, and miR-136. Each miRNA can participate in different diseases with different signal pathways, and each miRNA with different molecular mechanisms can also regulate the same disease for their inherent regulatory mechanism on PE, such as the miR-136, miR-494, and miR-495 genes [35], but not all of them can interact with circ_0055724. Through investigation and research, we found that the first three target miRNAs of circ_0055724 have never been studied in PE, while miR-136 has been reported in PE [34, 35]. It has laid a good theoretical and practical foundation for our study, making our research more rigorous and accurate. In this study, increased expression of miR-136 was observed during the current study in PE placental tissue samples, which is in accordance with the previous reports.

We used HTR-8/SVneo cells (ATCC® CRL-3271™) that are transfected trophoblasts and are widely used for the study of trophoblast biology and placental function [36, 37]. Overexpression of circ_0055724 resulted in increased viability and a greater number of migrating and invasive cells than control, while circ_0055724 silencing resulted in opposite effects. Overexpression of circ_0055724 in HTR-8/SVneo cells resulted in the inhibition of miR-136 expression while circ_0055724 silencing increased miR-136 expression. Interestingly, a negative correlation was observed between circ_0055724 and miR-136 in PE placental tissue, which is in accordance with the findings *in vitro*. Besides, overexpression of miR-316 decreased relative luciferase activity in WT HTR-8/SVneo cells but not in cells with circ_0055724-Mut. This confirms that circ_0055724 exerted its effect through the inhibition of hsa-miR-316. The role of miR-136 in the regulation of various biochemical pathways has been reported previously. It has been shown that a circular RNA, hsa_circ_0023404, acts as a miR-136 sponge in cervical cancer cells [38]. Another study indicated that a chondrocyte-derived circular RNA acts as a miR-136 “sponge” and regulated human cartilage degradation [39].

Another bioinformatics tool, Starbase, indicated the binding site of miR-316 and N-cadherin in trophoblast cells. miR-136 knockdown resulted in the increased expression of N-cadherin while miR-136 overexpression decreased the expression of N-cadherin in HTR-8/SVneo cells. It demonstrated that circ_0055724+miR-136 N-cadherin coexpression inversed the increased expression of N-cadherin caused by circ_0055724 overexpression alone. *In vivo* results indicated that PE placental tissue samples had decreased expression of N-cadherin. Hence, a positive correlation was observed between N-cadherin and circ_0055724 expression, while miR-136 and N-cadherin were negatively correlated. Lastly, increased cell viability, migration, and invasive capacity of HTR-8/SVneo cells caused by circ_0055724 overexpression were inhibited by circ_0055724+miR-136 coexpression or circ_0055724 overexpression but with N-cadherin knockdown.

N-cadherin is closely linked to cell migrative and invasive ability and can be a marker of cell epithelial-mesenchymal transition [40]. Previous studies have reported that N-cadherin promotes motility of cells in breast cancer [41], while N-cadherin exogenous expression induces cell migration and invasion of breast cancer cells [42]. Indeed, lncRNA SNHG5 regulates trophoblast cell proliferation, invasion, and migration via modulating the miR-26a-5p/N-cadherin axis [20], which further corroborates the role of N-cadherin in the regulation of trophoblast cells.

In general, circ_0055724 can act as a ceRNA to inhibit trophoblastic cell line HTR-8/SVneo's invasive and migratory abilities via the miR-136/N-cadherin axis, which is consistent with the previous roles of many circRNAs in PE as ceRNAs. For instance, upregulation of circRNA hsa_circ_0008726 in PE inhibits trophoblast migration, invasion, and EMT by regulating the miR-345-3p/RYBP axis [43]. Similarly, circ_0001438 participates in the pathogenesis of PE via the circ_0001438/miR-942/NLRP3 regulatory network [44]. As a novelty, our study is the first study to investigate the role played by circ_0055724 in PE, not only for its function but also for the intrinsic mechanism of its regulation. To conclude, this study provides an outline of how circ_0001438 plays a role in PE progression and may serve as a potential therapeutic target and a prognostic biomarker in PE.

In conclusion, for the first time, we reported that circ_0055724 exhibits a significant downregulated expression in PE tissues and could act as a promoter gene in PE. Our results found a new mechanism in the progression of PE which displayed that circ_0055724 could modulate the expression of N-cadherin through targeting the miR-136 in HTR-8/SVneo cells to regulate the proliferation, migration, and invasion of HTR-8/SVneo cells. These findings revealed that circ_0055724 could be a new therapeutic target for PE treatment.

## Data Availability

All supporting data of this work, which are not available in public because of the ethical restrictions, are available from the corresponding author upon request.

## Supplementary Materials

Supplementary Materials Figure S1: the relative expression of miR-136 in HTR-8/SVneo cells which cotransfected mimics-miR 136 and inh-miR-136. ^∗∗^*p* < 0.001.
