# Supplementary material for: circRNA circ_0055724 Inhibits Trophoblastic Cell Line HTR-8/SVneo's Invasive and Migratory Abilities via the miR-136/N-Cadherin Axis
Source: Dis Markers. 2022 Jun 22;2022:9390731. doi: 10.1155/2022/9390731 (PMC9242821; doi:10.1155/2022/9390731)
Supplement: Supplementary Materials — Figure S1: the relative expression of miR-136 in HTR-8/SVneo cells which cotransfected mimics-miR 136 and inh-miR-136. ∗∗p < 0.001. [file 9390731.f1.docx]

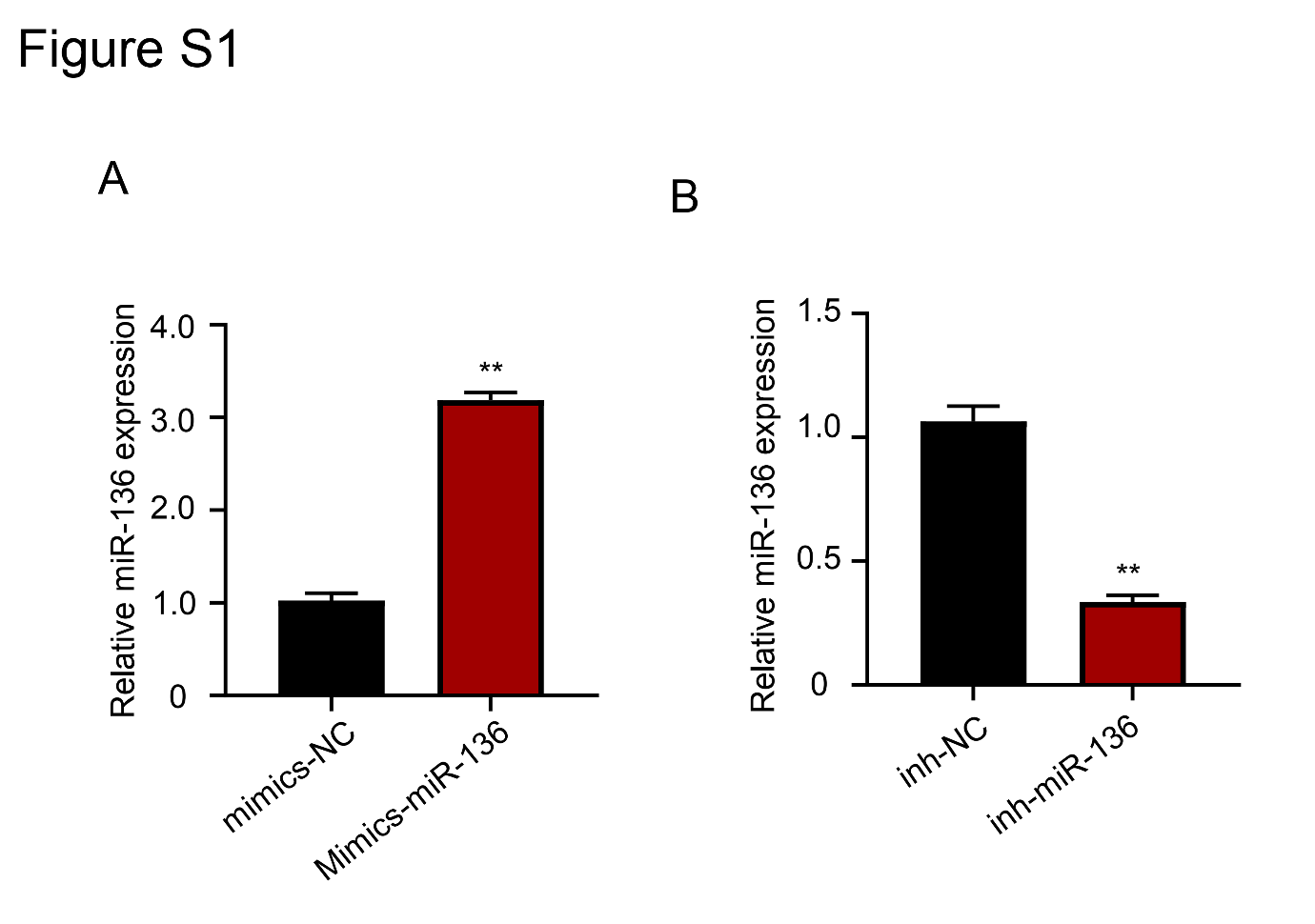


**Figure S1. The relative expression of miR-136 in HTR-8/SVneo cells which co-transfected mimics-miR 136 and inh-miR-136.** ^**^*P* < 0.001.
